# The Advantages of Capping Pulps of Teeth

**Published:** 1874-05

**Authors:** F. E. Howard

**Affiliations:** Genesee, N. Y.


### SELECTED ARTICLES.

---

### ARTICLE III.

### *The Advantages of Capping Pulps of Teeth.*

Read before the Seventh District Dental Society at Rochester, N. Y.

BY DR. F. E. HOWARD, GENESEE, N. Y.

It was allotted me this subject, that I might contribute my mite in behalf of the science of Dentistry, and this I most cheerfully do, and if it reflects one spark of light upon this important subject, I shall feel amply repaid for the little time spent in collecting these thoughts together.

About four years ago I made my first attempt upon capping an exposed pulp of a tooth. The subject was a young lady about eighteen, of a scrofula and inflammatory diathesis. The record of the case is as follows:

Miss M. presented herself for examination of the teeth, May 2nd, 1869. Her right lateral superior incisor was badly decayed, the pulp exposed, and bled from excavating. I filled with oxychloride of zinc alone, and dismissed my patient for a future sitting. In a few days she called. During her absence she had suffered considerably from pain in the tooth, and upon examination I determined to remove the filling. I found the pulp in a congested state. I decided to make an application of arsenic and extirpate the pulp. This was done, and the canal filled and the tooth saved by this method of treatment.

My second attempt was no more successful than the first, but this did not discourage me. The third proved a success; for one year after I had capped the exposed pulp in this case, I cut away the chloride of zinc filling and found the pulp alive. It had not in this length of time protected itself by a deposit of secondary dentine. I capped again and inserted a permanent gold filling. One year after the last operation the pulp was alive, and no better result could have been desired.

In this case the patient was of a sanguine lymphatic temperament.

Case 7 was that of a young man eighteen or nineteen years of age, of a sanguine temperament. The tooth was a right superior lateral incisor. The pulp was badly exposed in excavating, for I restored the last portion with a contour filling which was completed at the same sitting. This case comes under my observation often; I made a close examination of the condition of things but a short time ago, and I know that the tooth contains a live pulp that performs its functions with its associates. The case was treated in June, 1870.

Case 10 was that of a young man about eighteen, of a sanguine bilious temperament. The pulp was not exposed so that it bled. I made an application of Hill's stopping to the pulp, and filled with amalgam at the same sitting, and at the present day is a successful operation, after a test of over two years. This case is brought to your notice more particularly to show that success can be obtained with other materials besides oxychloride of zinc, but the latter is much preferred, for reasons that will be spoken of hereafter.

Case 15 was that of a lady forty years of age, of bilious temperament. The tooth was the first right inferior bicuspid. The pulp was badly exposed, and bled from excavating. I looked upon it as being rather a doubtful case. It was a distal approximate cavity, difficult of access on account of the backward inclination of the tooth.

I was not permitted to cut away into the grinding surface of the tooth as much as I should have done if it had been less sensitive. The pulp was capped with oxychloride of zinc and filled with amalgam immediately.

Two years after, the filling became loose and she presented herself again.

At this sitting the cavity was excavated almost to my entire satisfaction, though normally sensitive.

All of the oxychloride of zinc was removed, and the pulp was beautifully protected by a deposit of secondary dentine.

After properly excavating, the tooth was permanently filled with gold.

Case 20 proved a failure, but I am inclined to think it was because the cap was broken upon introducing the gold, as the zone of the exposure was considerable ; being a cavity upon the buccal surface of an under molar, it was difficult of approach and hard to control the flow of saliva.

I took some chance in this case as to what the result would be. I was quite of the opinion that I should not be crowned with success, and I was not ; for about one year after I found the pulp dead.

I was, however, successful in capping another pulp for the same patient.

Case 31 was that of a young lady twenty years of age, of a scrofula diathesis. The tooth was the left central superior incisor. This was considerably lopped over the right central, and in excavating I had to necessarily expose the pulp so that it bled. I capped in the usual way and filled with gold.

Eight months after, the filling became loose on account of the cavity not being shaped as I should have had it, were it not for the encroachment upon the pulp to too great an extent. The lady presented herself again, and at this sitting I shaped the cavity quite satisfactorily to myself, without removing all of the oxychloride of zinc, and refilled again. To-day the tooth performs its function as regularly as any of its neighbors. It was first capped February 3rd, 1871.

Case 35 was that of a young man aged twenty-two of bilious temperament. Tooth, first superior molar, proximate cavity. Capped and filled with amalgam at the same sitting.

Over two years after, decay had occurred, so that it was necessary to remove the filling and refill. I again exposed the pulp, capped as usual, and feel confident of success.

Case 36 was that of a little Miss fourteen years of age, of a sanguine bilious temperament. Tooth, first left superior molar, anterior proximate cavity. The pulp capped as usual and the cavity filled with gold at the same sitting.

A small cavity in the grinding surface presented itself, and should have been filled at the time, but she left town for school. On her return to my office, in one year and a half from that time, the decay had progressed so far that it had caused the destruction of part of the filling alluded to, and I deemed it advisable to remove the whole. The strong septum that divided the two cavities had become so much disorganized that I was compelled to work the two into a compound cavity. In doing this I found the pulp to be in a healthy condition, but the shape of the cavity and the surrounding circumstances prevented me from capping the second time, and I exceedingly regret to say I was obliged to extirpate the pulp and fill the canals.

Case 82 was that of a lady about twenty-two years of age, of nervous bilious temperament. In this case the pulp was exposed in the left superior cuspid. It was found somewhat congested. It was wounded in excavating, and, I think, relieved by it. I medicated with simply creasote for a few days. There seemed to be slight recision of the pulp. In excavating, a small chip got into the cavity or depression, and could not by any means be removed. I thought it would cause trouble by irritation if it was not removed, and I exposed the pulp more on the distal peripheral portion and excised a portion of it and removed at the same time the particle that was retained. I then bathed again with creasote and capped with oxychloride of zinc, and filled temporarily with Hill's stopping for two months. At the expiration of that time I filled permanently with gold. As yet the pulp remains healthy, after being subjected to this treatment over seven months ago.*

This case is noted to show that, though the pulp be accidently injured, it need not necessarily be lost.

Many other interesting cases might have been brought to your notice, but these will suffice to show some of the advantages of this method of treatment.

Gentlemen: These cases have been presented to your notice particularly, for I think they are marked cases of success and failure. During the last four years I have capped ninety-eight exposed pulps. Many of these cases have been capped under unfavorable circumstances, and yet my operations of this kind have been in a large percentage of cases successful.

Half of these cases have come under my observation months after the operation had been performed, and in many instances I have had the gratification of finding them alive and healthy over three years after they had been capped.

Of the cases seen after the operation had been performed, I consider I have only had six die. What the percentage of success will be with the other half is yet to be seen, but I have no reason to expect any worse results. What cause have I to look for a reaction after a month or a year's time? I cannot believe it. No; if bad results follow, I think they must come within a few weeks or months after the operation has been performed.

Allowing failures do occur, I think that a greater percentage of cases are satisfactory both to operator and patient when this method is pursued, than when the pulps are destroyed and the canals filled.

When we can be taught to shoot around a corner and hit the mark, we will be more successful in filling the canals of teeth; for how often do roots present themselves curved a short distance from the apex, that are impossible to fill well. If not filled to the very end, there is a receptacle left for

I have just tested this tooth carefully, and find the pulp still alive and healthy, after being capped sixteen months ago.

the retention of irritating matter, that will scarcely ever fail to do duty in that direction.

These things never developed themselves fully until the tooth had been extracted. I have seen instances where teeth have been filled by careful operation, that have done tolerable good service for years, that occasioned inconvenience at times since the tooth had been treated. Upon extracting such teeth after the patient had refused to endure the annoyance any longer (though trifling, perhaps, I may say,) what developed itself? A root that was curved at such an angle that it could not be filled perfectly. No one was to blame; an unhappy misfortune both for patient and operator.

Gentlemen, these are cases that have come within the observation of us all. They are not isolated cases, and I present them as an offset to failures in capping exposed pulps. I think there is more success with careful manipulation in capping than there is in extirpating and filling canals. No one will deny that if you are crowned with success in the former class of cases, you have done more for your patient than if you had been successful in the latter; for a tooth that performs its entire function is more useful and better able to meet the morbid influences brought to bear against it, than one that is partly devoid of vitality.

The editor of the *Dental Register* says: "It is a fact generally recognized by every dentist of much experience or close observation, that the integrity of the enamel and dentine of pulpless teeth pass away, with, however, varying degrees of rapidity, dependent upon the character of their structure, and their being modified by the original, together with the condition and force of the organizing and developing agencies at the time of their formation."

Usually teeth deprived of their pulp become in a comparatively short time friable, which is clearly shown by the readiness and frequency with which thin portions break away. This is the difficulty that generally follows the operation of filling such teeth.

This impairment of the enamel and dentine occurs because of the deprivation of nutrient supply; and it is not only exhibited in the manner referred to, but also by the increased susceptibility of the tooth substance to the action of decay producing agents.

The teeth vary exceedingly in the character of their structure; they have general features and aspects that are common to all, yet there is to every one an individually which is constituted by variation in structural details. This variation is palpable to the unaided vision in respect to form and size, but is quite as plainly marked in the minute structure under the microscope.

These variations are in the teeth, as in other structures or tissues, the occasion of ever varying susceptibilities, both in living and dead. The organization in some is so thorough, and the dentine and enamel so nearly perfect, as to resist any ordinary decay producing agents that are brought in contact with them, and sometimes to great extent even after devitalization, while in other cases so inferior are these structures that they are scarcely able to maintain their integrity when but slightly and only for a short time exposed to the feeblest decomposing agents, even when possessed of their normal vitality; and with such, devitalization is followed by rapid and utter dissolution.

Now there is an almost infinite variety of predisposition and susceptibilities between the extremes just presented, and the ability to apprehend and understand these conditions and susceptibilities, and in treatment to follow the indication, is an attainment of no mean order, and one that should be sought by every dentist.

The integrity of organized living tissues is maintained by the presence of vital principle, together with the process carried on by it, and as is already intimated, the hold of vitality upon the material part of tissue is exceedingly variable; in some holding a very firm grasp, and in others yielding its tenure at the first approach of danger. These things being true, the conclusion is forced upon us that the most acute and cultivated perception is requisite for the proper management of the teeth after they are attacked by disease.

Teeth, the pulp of which are destroyed, have no vitality, and receive no nutrition, and in common with all organized structure deprived of vitality, immediately begin to undergo deterioration, and sooner or later suffer entire dissolution.

With the teeth, however, the change may be materially modified by proper management, which consists in maintaining the most favorable conditions of the parts and tissues around about them, and excluding, so far as possible, all injurious agents and protecting the tooth at all points, so far as may be, from all mechanical violence, either in the way of strokes or undue attrition. By attentive observation and thought upon this subject, the educated dentist will not fail to perceive that pulpless teeth require the utmost care for their preservation for any considerable length of time; that the conservation of living is far easier than those devitalized, and that the perservation of pulp of the teeth is a matter of the utmost importance to those who desire to preserve their teeth in the best condition for the longest possible period.

The dentist who endeavors to cap pulp under favorable conditions has two chances of general success to one of extirpating and filling canals; for if you are not successful by the former method you may be by the latter.

It is anything but an easy matter to remove the filling from the canals of teeth, and if you are not successful in filling to the outer extremity of these canals you will hardly ever escape periosteal inflammation or an abscess, and if you are determined to overcome the difficulty you will have to remove the filling in order to remove the exciting cause of the trouble.

Show me the man who has no abscess formed from filling roots, and I will show you one who never had a failure in capping pulps.

If, after a time, you find that the pulp is dead, you need not remove the filling to amend difficulties, for you can almost always get better access to the canals by making another cavity in direct line with the axis of the tooth, than you can to treat the case from the cavity already filled ; for I will venture to say that in one hundred cases of exposed pulps, ninety of these will be upon proximate surfaces, and this is almost always an unsatisfactory introduction to the pulp canals. If you are not successful in saving the pulp of the tooth, you have hardly ever lost anything by the method of operation, for the approach to the canal in direct line with the axis of the tooth always compensates for the lost tooth structure. If you should be so unfortunate as to have to remove the filling in the third operation, you have gained much in a point of position. We cannot expect to be crowned with success in all cases in either mode of operating. We must look at the general average, and decide for ourselves which is the best treatment in the main, and under the existing circumstances. If you attempt to cap every exposed pulp, you find those diseased as well as healthy. You will not be successful with all, and should not condemn the practice.

I believe a great deal depends upon the manner in manipulating, and the kind of material used.

There certainly is a difference in the appearance and the general characteristics of the different preparations of oxychloride of zinc used for this purpose.

My manner of using this is first to bathe the pulp with creosote. I have used carbolic acid with good success. Then mix the oxychloride of zinc to about the consistency of cream, and with a small piece of spunk cut for the purpose, that can be nicely introduced into the cavity. I take this with plyers and dip into the prepared zinc and carry it directly to the exposed pulp and press very gently. I then take another piece of dry spunk and introduce this into the cavity, to absorb the excess of moisture in the oxychloride of zinc. Apply more oxychloride of zinc in the same manner and absorb the excess of moisture again.

By applying the preparation in this way there is scarcely any surplus of the material to be cut away. I consider only a thin layer of oxychloride necessary for success. I think that in some cases, where the whole cavity is filled with this material, bad results follow from the excess of hydrochlorate, which has a deleterious influence upon the pulps in some conditions.

By applying a small portion and absorbing the moisture from it, it does not act as so powerful an escharotic as it would otherwise. Any application of medicine to the animal economy must be in the right proportion in order to get the happy effect. An overdose of almost anything will work mischief. Hydro-chlorate of zinc is a powerful escharotic, and we could hardly expect satisfactory results by applying an excess of the acid to such a delicate organ as the pulp of a tooth.

I believe that of all the preparations used, oxychloride of zinc is the best, for it is porous to a certain extent, and if there is an exudation from the pulp it will be taken up by the oxychloride.

I consider that creosote or carbolic acid should always be applied to the pulp before introducing the oxychloride of zinc, for by its affinity for albumen and gelatine the pulp is protected in the form of a pellicle. There is an affinity between hydro-chlorate and the elements of the pulp, but the effect is not so pleasant.

From my experience in the results of this class of cases I can scarcely see how any progressive dentist can stick to the old method exclusively of extirpating pulps and filling canals, for really everything seems to me to be in favor of making an attempt at capping and preserving these useful organs in as perfect a condition as possible.—*Dental Miscellany*.

